# Superelastic and Ultra‐Soft MXene/CNF Aerogel@PDMS‐Based Dual‐Modal Pressure Sensor for Complex Stimuli Monitoring

**DOI:** 10.1002/advs.202502797

**Published:** 2025-04-07

**Authors:** Ao Wang, Zhenqiu Gao, Shaokuan Wu, Yihan Wei, Bohan Lu, Jia Shi, Lanyue Shen, Yina Liu, Xuhui Sun, Zhen Wen

**Affiliations:** ^1^ Institute of Functional Nano and Soft Materials (FUNSOM) Jiangsu Key Laboratory for Carbon‐Based Functional Materials & Devices Soochow University Suzhou 215123 P. R. China; ^2^ Department of Applied Mathematics, School of Mathematics and Physics Xi'an Jiaotong‐Liverpool University Suzhou 215123 P. R. China

**Keywords:** complex stimuli monitoring, dual mode, MXene aerogel, piezoresistive effect, pressure sensor, triboelectric effect

## Abstract

In the face of complex pressure stimuli, pressure sensor is required to sense the magnitude of static force and sensitive to transient mechanical stimuli. However, an individual sensing mechanism has difficulty meeting practical needs simultaneously. In this work, an MXene/cellulose nanofiber (CNF) aerogel@PDMS‐based dual‐modal pressure sensor is reported for complex stimuli monitoring. The aerogel‐based sensing material is fabricated through MXene nanosheets and CNFs. Aerogel ice crystals sublimate and then form a 3D porous structure during vacuum freeze‐drying. After attaching PDMS dilution, aerogels achieve >200 reversible compressions, and hysteresis energy is reduced by 57.8%. By utilizing both triboelectric and piezoresistive properties of MXene/CNF aerogel@PDMS, a dual‐modal pressure sensor is achieved. The triboelectric effect acquires high sensitivity of 26.95 kPa^−1^ under low pressure (3.46 Pa–3.32 kPa) and responds to vibrations up to 1000 Hz. On the basis of variable resistances of aerogels, the piezoresistive effect can be used to identify static pressures stably (167 kPa^−1^, 1.56–26.64 kPa). Combining two effects broadens the lower limit of high‐sensitivity monitoring, realizing static‐dynamic detection simultaneously and breaking the frequency limit of piezoresistive materials. Finally, the dual‐modal pressure sensor is demonstrated to monitor complex physiological and physical signals, such as pronunciation, gestures, and tone recognition.

## Graphical abstract

A triboelectric‐piezoresistive dual‐modal pressure sensor based on superelastic and ultrasoft MXene/cellulose nanofiber (CNF) aerogel@PDMS is fabricated. The sensor exhibits high sensitivity and stable sensing performance via a dual mechanism. Through the effective combination of these two mechanisms, comprehensive monitoring of complex stimuli can be achieved, overcoming the response frequency limit of traditional materials.

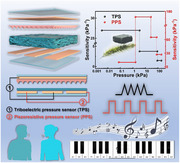

## 1 Introduction

As important media for monitoring various environmental stimuli, pressure sensors play important roles in the Internet of Things,^[^ ^1^ ^]^ e‐skins,^[^ ^2^ ^]^ wearable electronics,^[^ ^3^ ^]^ and human‒machine interactions.^[^ ^4^ ^]^ In the face of complex pressure stimuli, the pressure sensor should sense static forces and respond sensitively to transient mechanical stimuli. In recent years, piezoresistive,^[^ ^5^ ^]^ piezoelectric,^[^ ^6^ ^]^ triboelectric,^[^ ^7^ ^]^ and capacitive^[^ ^8^ ^]^ pressure sensing mechanisms have been proposed. However, when only relying on an individual sensing mechanism, pressure sensors face problems such as incomplete signal conversion and missing information. At present, many dual‐modal pressure sensors have emerged to realize the comprehensive monitoring of pressure stimuli.^[^ ^9^, ^10^, ^11^, ^12^ ^]^ According to the combination mode, one combines different sensing elements into a platform in a 3D space.^[^ ^13^, ^14^, ^15^ ^]^ For example, Kong et al.^[^ ^15^ ^]^ used an elastomer to transition the normal force to the transverse expansion force, achieving a static and dynamic signal response in the same dimension. This type of dual‐modal sensor is easy to manufacture and compact but has high power consumption and signal crosstalk problems. The other method involves fusing two complementary and compatible sensing mechanisms to manufacture dual‐modal sensor devices.^[^ ^11^, ^16^, ^17^ ^]^ This can generally reflect static and dynamic signals at the same time, and as the frequency increases, the signal changes from static to dynamic. However, the performance of the sensor material and the accuracy of the time and space synchronization of the sensor are especially high. Therefore, there is an urgent need for multifunctional materials with excellent electrical conductivity and good mechanical properties to promote the integration of different pressure‐sensing mechanisms to achieve more comprehensive monitoring.

The unique 3D structure of aerogels can provide a conductive framework that provides excellent electrical performance for flexible electronic devices such as piezoresistive sensors,^[^ ^18^ ^]^ supercapacitors,^[^ ^19^ ^]^ and nanogenerators.^[^ ^20^ ^]^ The adjustable contact site structure and high porosity allow real‐time adjustment of the aerogel under external pressure resistance.^[^ ^21^ ^]^ In addition, compared with traditional 2D structural materials, 3D porous materials can increase the density of the transmitted charge, thus improving the electrical output performance of the entire electronic device.^[^ ^22^, ^23^, ^24^ ^]^ Currently, aerogels for pressure sensing include materials such as graphene,^[^ ^25^ ^]^ MXene,^[^ ^26^ ^]^ carbon nanotubes,^[^ ^27^ ^]^ and conductive polymers.^[^ ^28^ ^]^ Among them, MXene is 2D metal carbides and nitrides with high carrier mobility, good electrical conductivity, and good mechanical strength.^[^ ^29^, ^30^, ^31^ ^]^ However, owing to the large spacing between MXene layers and the relatively weak van der Waals forces between adjacent pieces, the brittleness of MXene‐based aerogels is relatively high, and irreversible compressibility easily occurs. Nanofibers can support MXene between layers and improve their mechanical properties.^[^ ^19^, ^32^, ^33^ ^]^ In addition to the aerogel pore wall, the conductive fibers attached to the MXene can provide more conductive access when compressed.

In this work, an MXene/cellulose nanofiber (CNF) composite aerogel was prepared via the vacuum freeze‒drying method. Then, PDMS was used as a reinforcing agent to attach to the pore surface of the aerogel, and superelastic and ultrasoft MXene/CNF aerogel@PDMS was obtained. The properties and potential applications of dual‐modal pressure sensors using aerogels as triboelectric tribo‐layers and piezoresistive sensitive layers are explored. MXene nanosheets and CNFs complement each other, making the MXene/CNF aerogel have a 3D porous structure with line‐surface support. Under the attachment of PDMS with excellent elasticity and stability, the porous and low‐density aerogel has an ultralow Young's modulus (158 Pa), good compressive resilience (200 th), and fatigue resistance (3000 cycles). Therefore, the MXene/CNF aerogel@PDM‐based dual‐modal pressure sensor has excellent sensing performance. Owing to the complementary triboelectric and piezoresistive properties, the lower limit of the highly sensitive interval is effectively broadened, static and dynamic signals are comprehensively monitored, and the frequency limit of the response of piezoresistive materials is broken. In addition, we explored the versatility of the MXene/CNF aerogel@PDMS‐based dual‐modal pressure sensor for detecting human movement and physical signals, such as pronunciation, gestures, and tone recognition.

## 2 Results and Discussion

### 2.1 Device Structure Design and Characterization of Materials

**Figure 1:**
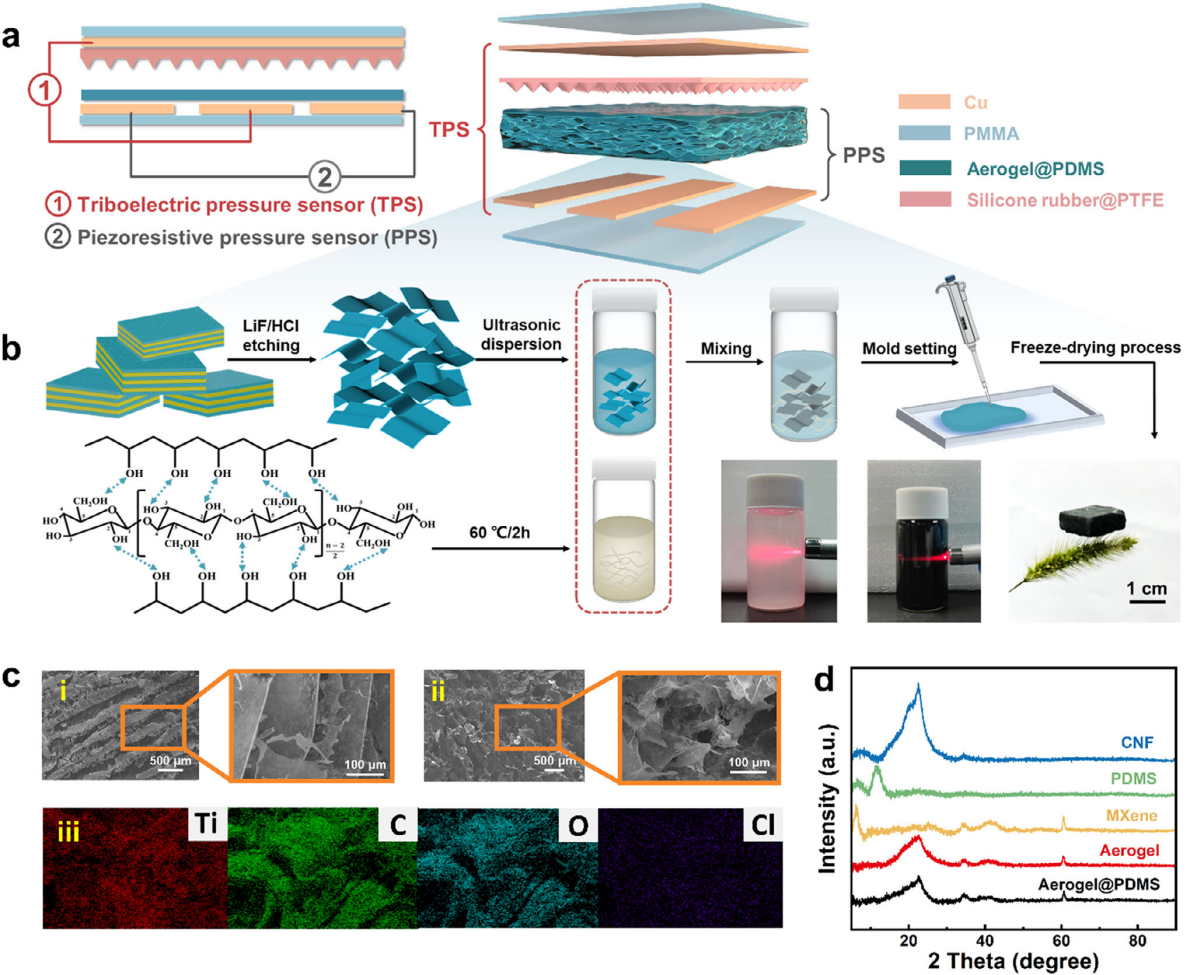
MXene/CNF aerogel@PDMS‐based dual‐modal pressure sensor. a) Structural diagram of dual‐modal pressure sensor; b) Preparation of MXene/CNF aerogel; c) SEM image and elemental mapping of aerogel‐10; d) XRD pattern.

**Figure** **1**a shows a schematic diagram of the device structure of the MXene/CNF aerogel@PDMS‐based dual‐modal pressure sensor. The aerogel acts as the triboelectric tribo‐layer and piezoresistive force‐sensitive layer simultaneously. The bottom copper electrode adopts a three‐separation structure to realize dual‐modal pressure sensing via triboelectric and piezoresistive mechanism. Among them, the TPS adopts the contact separation mode, and the electrodes are the top electrode and the bottom middle electrode. The electrodes of the PPS are on either side of the bottom electrodes. The preparation process of the MXene/CNF aerogel is shown in Figure 1b. The MXene nanosheet suspension, cellulose nanofiber suspension, and PVA solution were used as the precursor solutions of the aerogel. An ultralow‐density MXene/CNF aerogel was obtained by vacuum freeze‐drying. Then, the MXene/CNF aerogel was immersed in the PDMS diluent for 10 s, removed, and dried to obtain the MXene/CNF aerogel@PDMS. The size of the MXene nanosheets is approximately a few hundred nanometers (Figure S1a, Supporting Information), which are easy to stack. CNFs have good flexibility and mechanical support. According to the SEM images (Figure 1c‐i,ii), their combination results in a highly entangled, overlapping, 3D structure containing many micrometer‐ or nanometer‐scale pores. The elemental analysis of the aerogel (Figure 1c‐iii) revealed that the main elements included Ti and C in the MXene and C and O in the CNF. Cl is present in tiny amounts in the residue of the CNF preparation.

The XRD pattern is used to analyze the crystal structure of the obtained material, as shown in Figure 1d and Figure S1b (Supporting Information). After etching the Al layer, the diffraction peak of the (002) crystal plane of MXene at 2θ = 6.3° significantly shifts to the left compared with that of Ti_3_AlC_2_, indicating that its layer spacing increased.^[^ ^34^ ^]^ The diffraction peak of the (200) crystal plane of CNF at 2θ = 22° is relatively sharp, indicating that its crystallinity is high.^[^ ^35^ ^]^ As a high polymer, the diffraction peak at 2θ = 11.5° indicates that PDMS has a certain degree of crystallinity,^[^ ^36^ ^]^ which is helpful for determining the mechanical properties of the aerogel. Finally, the MXene/CNF aerogel and MXene/CNF aerogel@PDMS samples exhibited diffraction peaks at 2θ = 22.5° and 2θ = 60.5°, respectively.

### 2.2 Characterization of the Mechanical Properties of Aerogels

**Figure 2:**
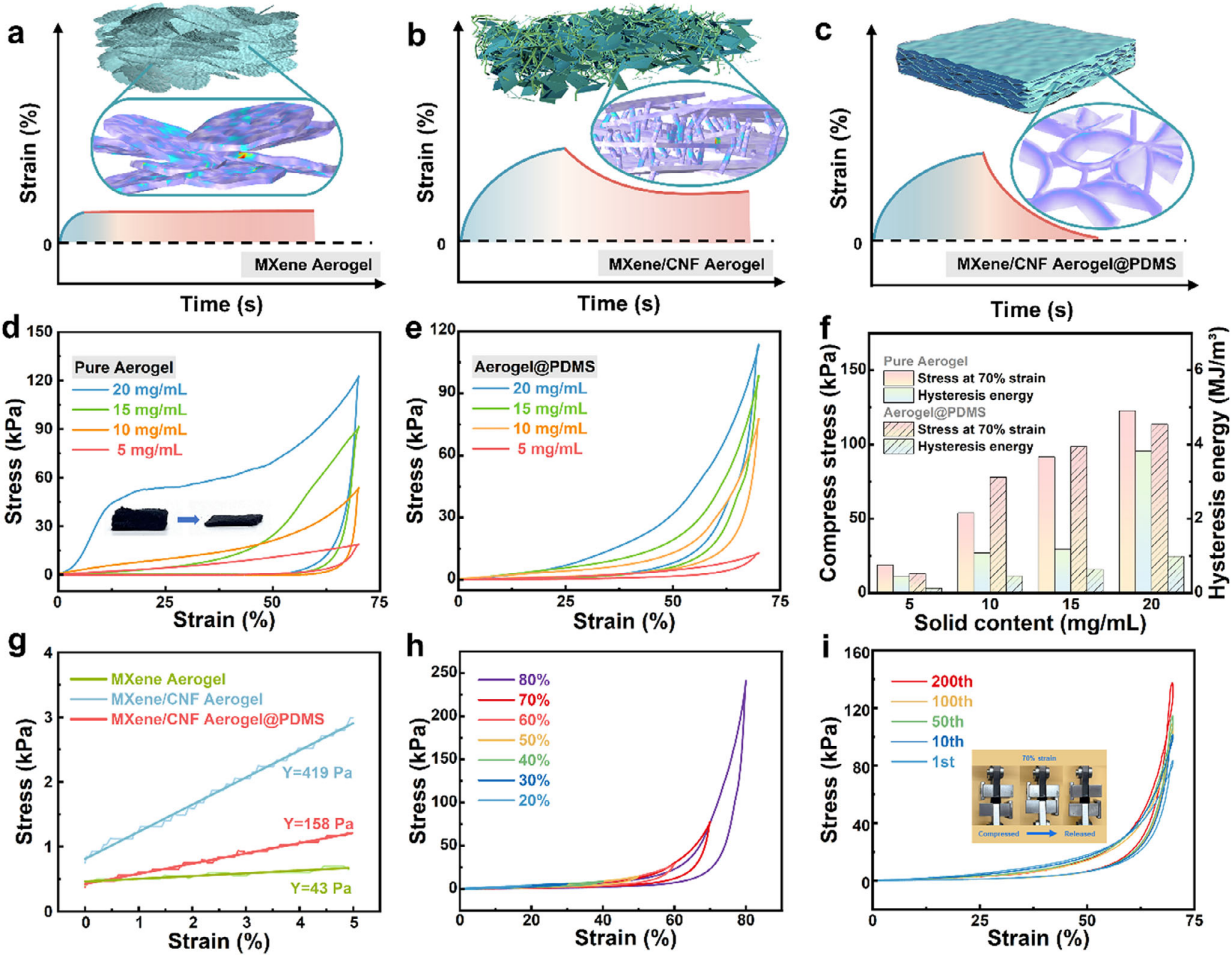
Mechanical properties of aerogels. Microstructure diagram and strain recovery analysis of a) MXene aerogel, b) MXene/CNF aerogel, and c) MXene/CNF aerogel@PDMS. d) Stress‒strain curve of the MXene/CNF aerogel. e) Stress‒strain curve of MXene/CNF aerogel@PDMS. f) Compressive stress and hysteresis energy at 70% strain. g) Young's moduli of MXene aerogel, MXene/CNF aerogel, and MXene/CNF aerogel@PDMS. h) MXene/CNF aerogel@PDMS stress‒strain curves for different strains. i) MXene/CNF aerogel@PDMS compression cycle.

By combining the simulation results of the microstructure force distributions of the MXene aerogel, MXene/CNF aerogel, and MXene/CNF aerogel@PDMS (**Figure** **2**a–c) and the uniaxial compression test results (Figure 2d,e), the aerogel composed of MXene nanosheets was found to be composed mainly of 2D nanosheets stacked to form a 3D aerogel structure, which easily adheres to each other and lacks longitudinal support points in the process of compression (Figure 2a). Therefore, the MXene aerogel has an ultralow Young's modulus of only 43 Pa (Figure 2g), and its ultrasoft properties make it poor in mechanical strength and unable to rebound for a long time after compression. After the addition of CNF, the MXene/CNF aerogel formed a structure with overlapping lines and planes. The high mechanical strength of the CNFs provided strong longitudinal support for the compression process (Figure 2b), which made it difficult for the aerogel to collapse. The Young's modulus of the MXene/CNF aerogel increased ≈10‐fold (419 Pa) because of the substantial increase in mechanical strength. However, the resilience after compression needs to be further improved, and the compressive hysteresis energy is still high (Figure 2f). As a polymer, PDMS has good flexibility and plasticity, making it easy to form various shapes and structures. The MXene/CNF aerogel was immersed in uncured PDMS‐n‐hexane solution and then quickly dried, leaving a small amount of PDMS (as a reinforcing agent) on the inner surface of the abundant pore pairs of the MXene/CNF aerogel to increase the toughness of the 3D frame of the MXene/CNF aerogel. Therefore, the MXene/CNF aerogel@PDMS not only retains the good mechanical strength of the MXene/CNF aerogel but is also is ultrasoft (158 Pa) and superelastic after compression. According to the uniaxial compression test results, the compression performance of the MXene/CNF aerogel@PDMS composite was greatly improved, and the compression hysteresis energy was reduced by >50%. In addition, the good chemical stability of PDMS also contributes greatly to the long‐term storage stability of aerogels in air.

The mechanical properties of aerogels with different solid contents ranging from 5 to 20 mg mL^−1^ were subsequently investigated. Owing to the low solid content of aerogel‐5, its mechanical strength is insufficient, and the stress is <30 kPa even under a large strain of 70%, so the monitoring range of aerogel‐5 used for pressure sensing is too small. With increasing solid content, the compressive stress of the aerogel increased to 122 kPa, but the increase in solid content also resulted in an increase in the density of the aerogel, a reduction in the number of pores and a reduction in size (Figure S2, Supporting Information). According to the compression strain curve of the MXene/CNF aerogel@PDMS composite, PDMS had the greatest improvement in performance on aerogel‐10. Then, uniaxial compression tests of 10 mg mL^−1^ aerogel with different compression degrees were carried out. The stress reached 242 kPa under 80% strain, and the compressive hysteresis energy also increased with increasing strain (Figure S3a, Supporting Information). In addition, aerogel‐10 rebounds well after being compressed 200 times under 70% strain (Figure 2i), and the compressive hysteresis energy eventually stabilizes at 0.34 MJ m^−^^3^ (Figure S3b, Supporting Information).

### 2.3 Sensing Performance of the MXene/CNF Aerogel@PDMS‐Based Dual‐Modal Pressure Sensor

**Figure 3:**
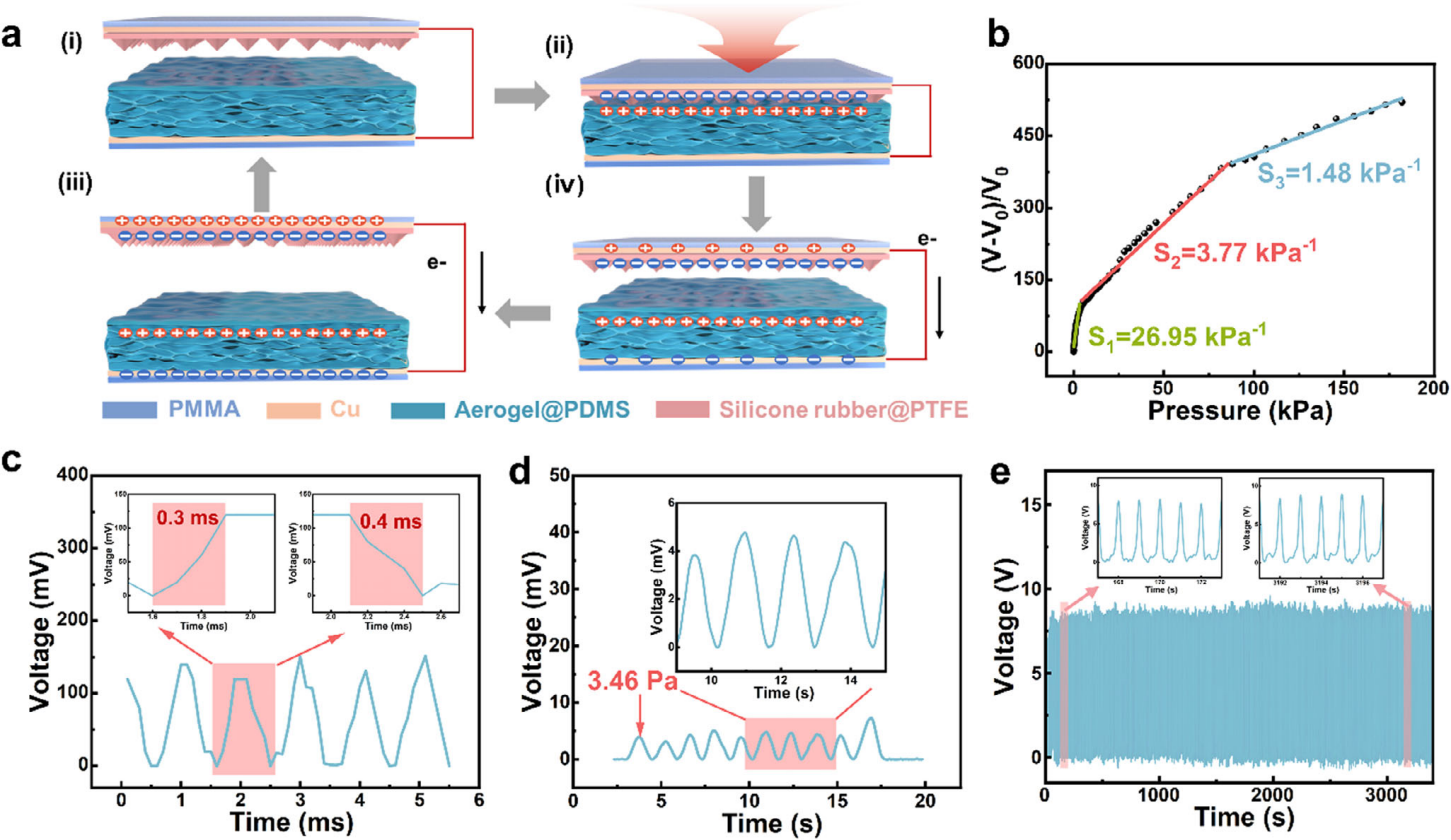
Triboelectric sensing performance of the MXene/CNF aerogel@PDMS‐based dual‐modal pressure sensor. a) Working mechanism of TPS. b) Pressure sensitivity of TPS. c) Response/recovery time of TPS. d) Minimum detection limit of TPS. e) Triboelectric signal stability.

Through triboelectric and electrostatic induction, the TPS can convert external pressure stimuli into electrical signals. **Figure** **3**a‐i–iv) shows the working mechanism of the TPS, which adopts the contact‐separation mode. Without external pressure, the aerogel layer and the silicone rubber@PTFE layer are in a static state without contact (Figure 3a‐i). When a pressure stimulus is applied, the aerogel layer and the silicone rubber@PTFE layer begin to contact each other, and owing to the opposite triboelectric properties of the two materials, opposite tribocharges are generated on the aerogel and the silicone rubber@PTFE surface (Figure 3a‐ii). When the pressure stimulus is removed, the compressed part begins to separate due to the good resilience of the aerogel, and inductive charges are generated in the upper and lower electrodes, resulting in charge transfer and output voltage signals, respectively (Figure 3a‐iii). Until the tribo‐layer is separated to the maximum gap, the free electrons are no longer transferred to restore the initial electrostatic equilibrium (Figure 3a‐iv). In this way, the pressure signal is converted into an electrical signal to achieve pressure sensing.

The triboelectric voltage output of the MXene/CNF aerogel@PDMS composite with 10 common tribomaterials was tested (Figure S4a, Supporting Information). According to previously reported work,^[^ ^37^ ^]^ a microstructural design on the surface of the tribo‐layer can effectively improve the sensitivity of the triboelectric pressure sensor. To obtain greater voltage output and convenient structural design, silicone rubber and PTFE particles are selected as another tribo‐layer. The preparation process of silicone rubber@PTFE is shown in Figure S5, Supporting Information. PTFE particles with a particle size of ≈5 µm were evenly dispersed in silica gel and coated on the acrylic template printed by a laser, which was cured and removed for use. According to the different proportions of silicone rubber@PTFE particles used for the triboelectric voltage output with aerogel (Figure S4b, Supporting Information), the proportion of silicone rubber@PTFE particles was 10:2. Then, the pressure sensing performance of the TPS is explored by controlling the relative voltage change (*∆V/V_0_*) under different pressures via a manometer. From the perspective of mechanical properties, aerogel‐5 is excluded as the choice of dual‐modal pressure sensing material. Among the triboelectric pressure sensing properties of aerogel‐10, aerogel‐15, and aerogel‐20 (Figure S6a, Supporting Information), aerogel‐10 stands out. Then, through the same structure, aerogel‐10 and copper were used as tribolayers to test the pressure‐sensing performance (Figure S6b, Supporting Information). The results show that the sensitivity of the device with aerogel is much greater than that of the device without aerogel. This is because the aerogel has an ultralow Young's modulus (Figure 2g) and good compression performance (Figure 2e), and there are many internal pores, which can be continuously compressed under increasing external pressure so that the distance *d* of the contact separation process continuously increases in space. According to Equations (1) and (2), the *∆V* of an aerogel‐based TPS can have space for continuous increase, so the sensitivity is much greater than that of a copper‐based TPS.

(1) $$V_{oc}=\frac{\sigma \cdot d}{\varepsilon_{0}}$$

(2) $$\Delta V=V-V_{0}$$

(3) $$S=\frac{\Delta V/V_{0}}{\Delta P}=\frac{\sigma_{0}}{\varepsilon_{0}}\cdot \frac{d}{Y}$$

where *V_oc_* is the open‐circuit voltage of the TPS; *σ* is the surface charge density; ε_0_ is the dielectric constant of air; *d* is the distance of contact separation; *S* is an abbreviation for the defined sensitivity; *∆V* indicates the voltage difference; and *Y* is Young's modulus.

The relationship between *∆V/V_0_* and pressure in Figure 3b reflects the stress sensitivity (*S*) of the TPS. The TPS has high sensitivity over a small pressure range, reaching a maximum of 26.95 kPa^−1^ in the range of 3.46 Pa–3.32 kPa. The TPS sensitivity is highly consistent with the strain curve of silicone rubber@PTFE (Figure S6c, Supporting Information). According to the literature,^[^ ^37^ ^]^ the sensitivity of a triboelectric pressure sensor is determined by the Young's modulus (*Y*) of the interlayer material and the distance *d* between the tribolayers, as shown in Equation (3). Therefore, the sensitivity curve of the TPS can be modeled into three parts, namely, the low‐pressure region, middle‐pressure region, and high‐pressure region, according to Young's modulus variation trend of silicone rubber@PTFE. The TPS demonstrates rapid response and recovery capabilities, with response/recovery times of only 0.3/0.4 ms (Figure 3c). When a small‐range manometer is used to apply a small‐pressure stimulus, the TPS can output the same voltage over multiple small‐pressure stimuli of just 3.46 Pa, demonstrating its stability and accuracy under tiny pressures (Figure 3d). In addition, because of the excellent tribomechanical properties of the aerogel, the TPS still has a stable voltage output after >3000 contact‐separation cycles, as shown in Figure 3e. After 20 days of TPS in air, the triboelectric voltage almost disappears (Figure S7, Supporting Information), which also provides strong support for the use of TPS in practical sensing applications.

**Figure 4:**
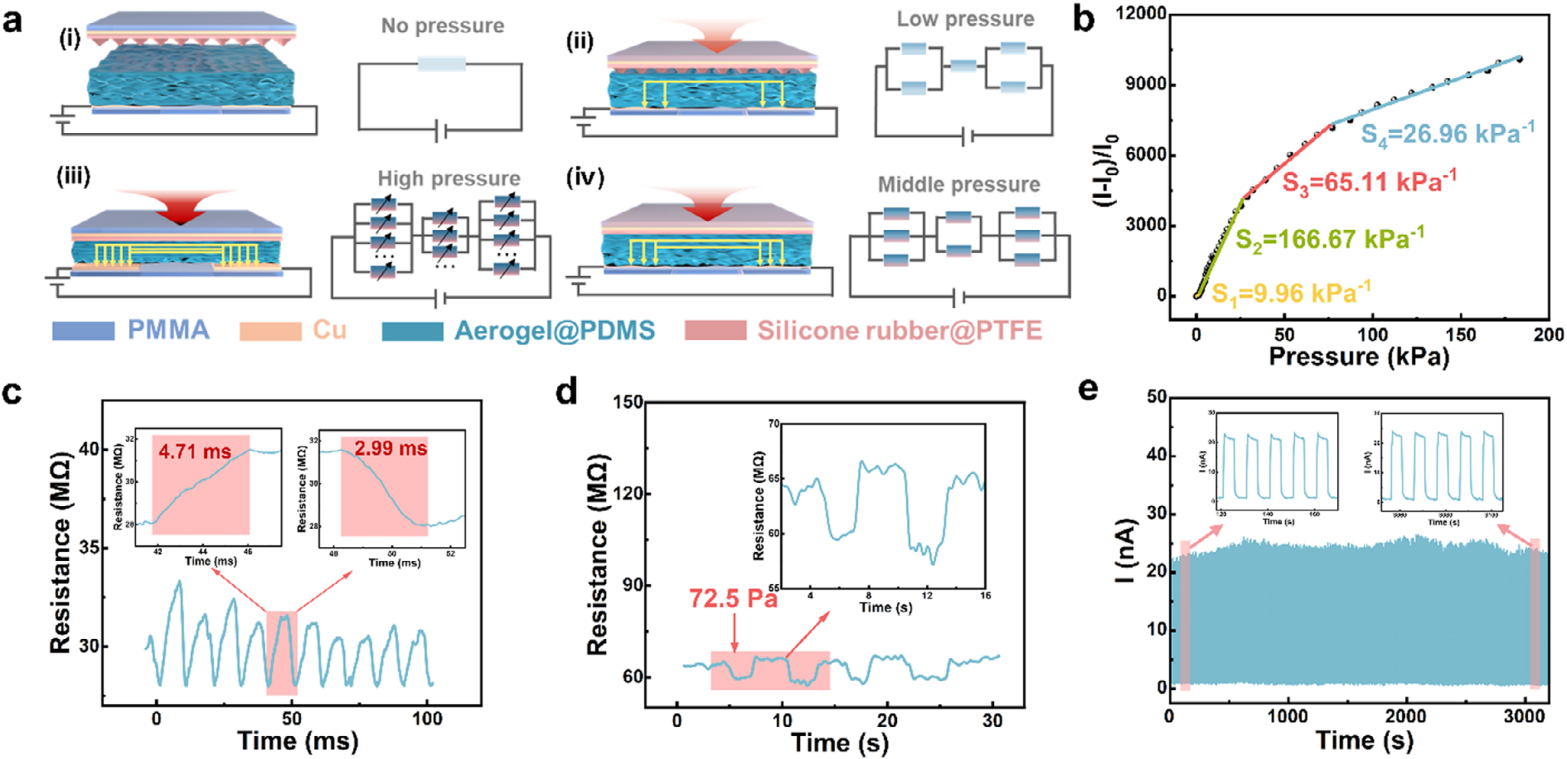
Piezoresistive sensing performance of the MXene/CNF aerogel@PDMS‐based dual‐modal pressure sensor. a) Working mechanism of PPS. b) Pressure sensitivity of the PPS. c) Response/recovery time of PPS. d) Minimum detection limit of PPS. e) Piezoresistive signal stability.

The working mechanism of PPS is shown in **Figure** **4**a. On the basis of the compressible strain characteristics of the aerogel, the resistance changes under pressure stimulus to achieve pressure sensing such that the pressure stimulus is converted into a resistance signal. First, in the state of no pressure stimulus, the current in the circuit is small because of the large initial resistance of the composite aerogel (Figure 4a‐i). Then, in the low‐pressure stage, because the pressure above is conducted through the micro cone of silicone rubber@PTFE, the strain of the aerogel is from the point to the surface, so the resistance change is not obvious in this stage (Figure 4a‐ii). When the applied pressure is further increased (Figure 4a‐iii), many pores are compressed, and the compressive strain of the aerogel rapidly increases. When the pressure continues to increase (Figure 4a‐iv), the compression degree of the aerogel is >60%, the internal fibers are compressed, the strain space is reduced, and the resistance change is also reduced.

The PPS pressure sensing performance is explored by measuring the relative current change (*∆I/I_0_*) at a fixed voltage (1 V). The good linear relationship of the current‒voltage (I‒V) curve indicates that MXene/CNF aerogel@PDMS has stable electrical characteristics (Figure S8, Supporting Information). For the piezoresistive pressure sensing performance of aerogel‐10, aerogel‐15, and aerogel‐20 (Figure S9a, Supporting Information), the variable range of aerogel resistance increases with increasing solid content, causing the upper limit of *∆I* to increase. According to Equation (4), the sensitivity *S* also increases. However, the compression hysteresis energy of the high solid‐content aerogel remained high (Figure 2f), so the signal stability was low (Figure S9b, Supporting Information). Therefore, according to the comprehensive consideration of the mechanical and electrical properties, aerogel‐10 was also selected as the piezoresistive force‐sensitive layer.

(4) $$S=\frac{\Delta I/I_{0}}{\Delta P}$$

where *∆I* refers to the change in current, *I_0_* is the initial current of the aerogel at a voltage of 1 V, and *∆P* is the change in pressure.

The relationship between *∆I/I_0_* and pressure in Figure 4b reflects the stress sensitivity of the PPS. The PPS sensitivity first increases but then decreases, which conforms to the equation *y* = − 140 + 202.9*x* − 2.17*x* ^2^ + 0.012*x* ^3^ − 0.00002*x*4 (Figure S10a, Supporting Information). After linear fitting, a maximum of 167.67 kPa^−1^ is achieved in the pressure range of 1.56–26.64 kPa because of the gradual improvement in the conductive path of the aerogel during the initial compression stage and the relatively low modulus. With further compression, the aerogel structure becomes dense, resulting in a dramatic increase in the modulus (Figure S10b, Supporting Information). Owing to the aerogel's excellent compressive elasticity, PPS has fast response and recovery capabilities, with a response time of 4.71 ms and a recovery time of 2.99 ms at ≈15% ∆R/R_0_ (Figure 4c). Five grams of weight are placed on the PPS several times to measure the resistance response at tiny pressures. As shown in Figure 4d, the resistance change maintains the same amplitude in repeated tests, indicating its stability and accuracy under tiny pressures. In addition, the aerogel's excellent fatigue resistance endows PPS with stable pressure‐sensing performance during multiple compression runs. As shown in Figure 4e, the current at a fixed voltage of 1 V always changes regularly and does not decrease significantly after >3000 cycles. In addition, after 20 days of placing PPS in air, the current can still maintain a regular change (Figure S11, Supporting Information), which indicates that PPS is reliable in practical sensing applications.

### 2.4 Application of the Dual‐Modal Pressure Sensor

**Figure 5:**
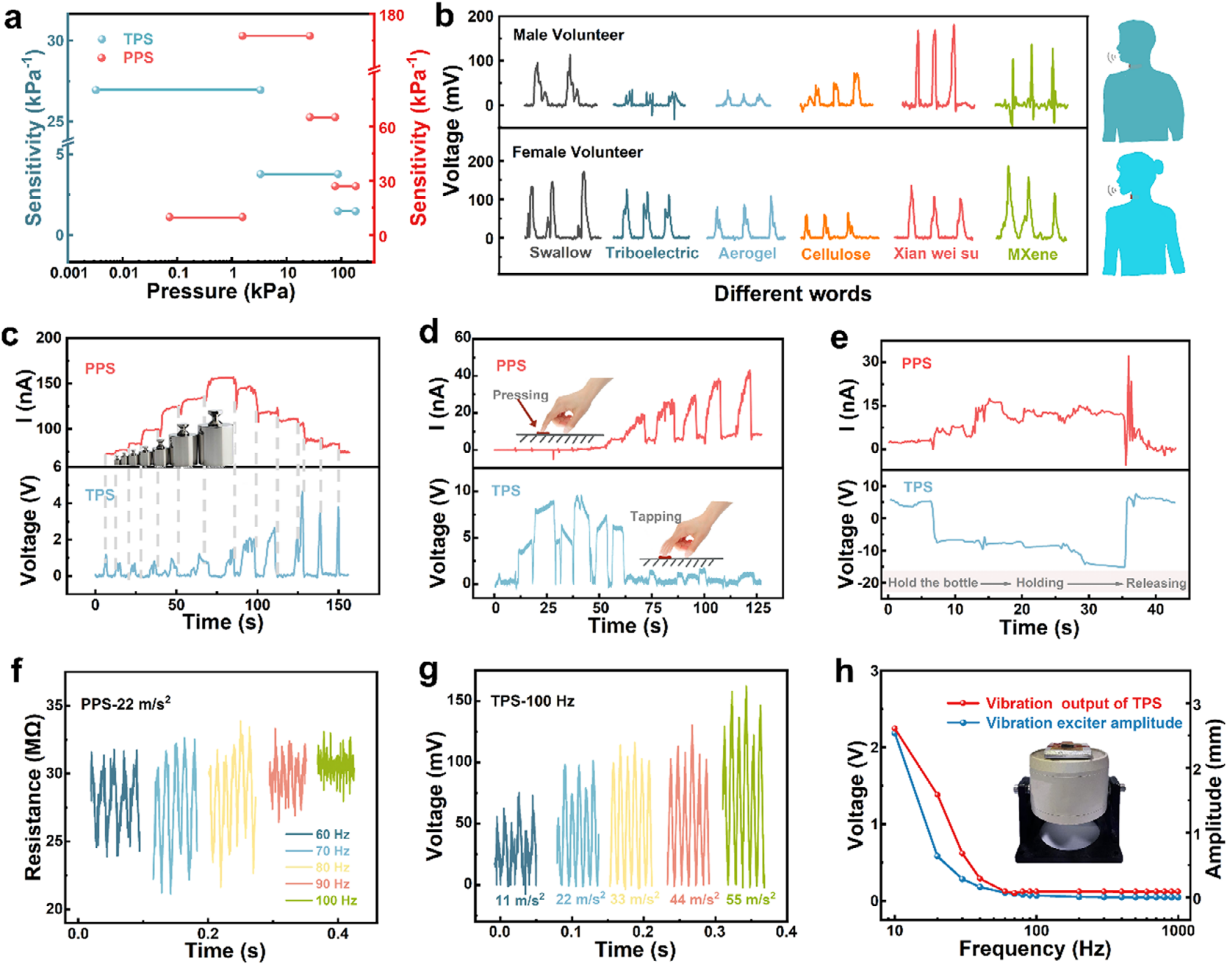
Practical application of the MXene/CNF aerogel@PDMS‐based dual‐modal pressure sensor. a) Sensitivity and detection range of the dual‐modal pressure sensor. b) Attaching to a human throat for monitoring different words. c) Testing of increasing or decreasing weights. d) Monitoring different levels of gestures: long pressing and quick tapping. e) Monitoring various processes of holding bottles. f) 60–100 Hz pressure sensing of PPS. g) 100 Hz pressure sensing of TPS. h) Relationships of the vibration exciter amplitude and triboelectric voltage output with frequency.

As shown in **Figure** **5**a, the dual‐modal pressure sensor has a wide detection range and high sensitivity. This sensor is lightweight and convenient, so it can detect various complex stimuli in daily life. Because the electrode material and sensing material of the dual‐mode pressure sensor are flexible, it can be closely fitted with various parts of the human body. In the process of monitoring human physiological signals, pressure information can be effectively transmitted to achieve efficient detection. In the process of application, we use a flexible PC film on the outside of the device for packaging to ensure reliable signal stability. For example, attaching a sensor to a volunteer's throat via medical tape can monitor what the volunteer says. We monitored the swallowing behavior and speaking five words of male and female volunteers. The sensor can effectively distinguish between different types of swallowing and speech (Figure 5b). According to the obtained voltage waveform, it can be found that the waveform can be repeated well for the same word spoken by the same volunteer. For different volunteers, owing to the physiological differences between male and female volunteers, such as the morphology of Adam's apple, skin state, and pronunciation characteristics, two volunteers can effectively distinguish the same word. By combining big data and machine learning, it may be possible to calculate and analyze sounds from waveforms for speech recognition. Thus, by using the difference between triboelectric and piezoresistive signals, static and dynamic signals can be recognized effectively.

As shown in Figure 5c, different weights are successively increased above the device and then decreased in turn. The process of increasing and decreasing pressure can be observed from the piezoresistive signal. The contact separation process caused by taking the weight can be seen from the triboelectric signal. Moreover, this dual‐modal pressure sensor can accurately identify small tapping gestures with the help of the high sensitivity of the TPS. Then, PPS can respond to gestures of pressing with different forces that do not separate for a long time (Figure 5d). For a more complete demonstration, we also attach the device to the inside of the palm to monitor the entire process from picking up the glass to putting it down (Figure 5e), which demonstrates that the sensor can be used to detect both static and dynamic pressure stimuli.

In addition, for most piezoresistive pressure sensors, which use elastomers for pressure sensing, monitoring signals at frequencies >100 Hz is challenging. According to the dynamic mechanical analysis results of MXene/CNF aerogel@PDMS (Figure S12a, Supporting Information), the storage modulus of MXene/CNF aerogel@PDMS begins to be lower than its loss modulus from 70 Hz onward. This means that as the frequency increases, the aerogel begins to change from an elastomer to a viscous material, and the compressive resilience does not match the applied pressure, so there will be no piezoresistive response.^[^ ^38^ ^]^ Therefore, for the PPS, when the signal frequency provided by the vibration exciter reaches 100 Hz, there is no response to the pressure signal (Figure 5f). On the other hand, the TPS can very clearly distinguish between 100 Hz signals of different accelerations provided by the vibration exciter (Figure 5g), up to 1000 Hz. The relationship between the vibration amplitude of the vibration exciter and the acceleration and frequency is shown in Equation (5), so under fixed acceleration, the amplitude decreases with increasing frequency.

(5) $$a\;={\left(2\pi f\right)}^{2}d$$

where *a* is the acceleration of vibration of the vibration exciter; *f* is the frequency of vibration of the vibration exciter; and *d* is the vibration amplitude of the vibration exciter.

Moreover, the magnitude of the triboelectric voltage is related to the compression distance *d* (i.e., the amplitude of the vibration exciter). Therefore, the triboelectric voltage and frequency relationship will have the same variation tendency as the amplitude and frequency relationship of the vibration exciter. When the frequency is increased to 1000 Hz, the triboelectric voltage output is ≈120 mV (Figure 5h). In addition, as shown in Figure S12b,c, we also apply a prestress by placing weights above the device and find that the PPS can effectively demonstrate the response under different prestresses at 60 Hz. The response of the TPS under different prestresses is not obvious. This also reflects the complementary monitoring effect of the dual‐modal pressure sensor on static and dynamic signals.

**Figure 6:**
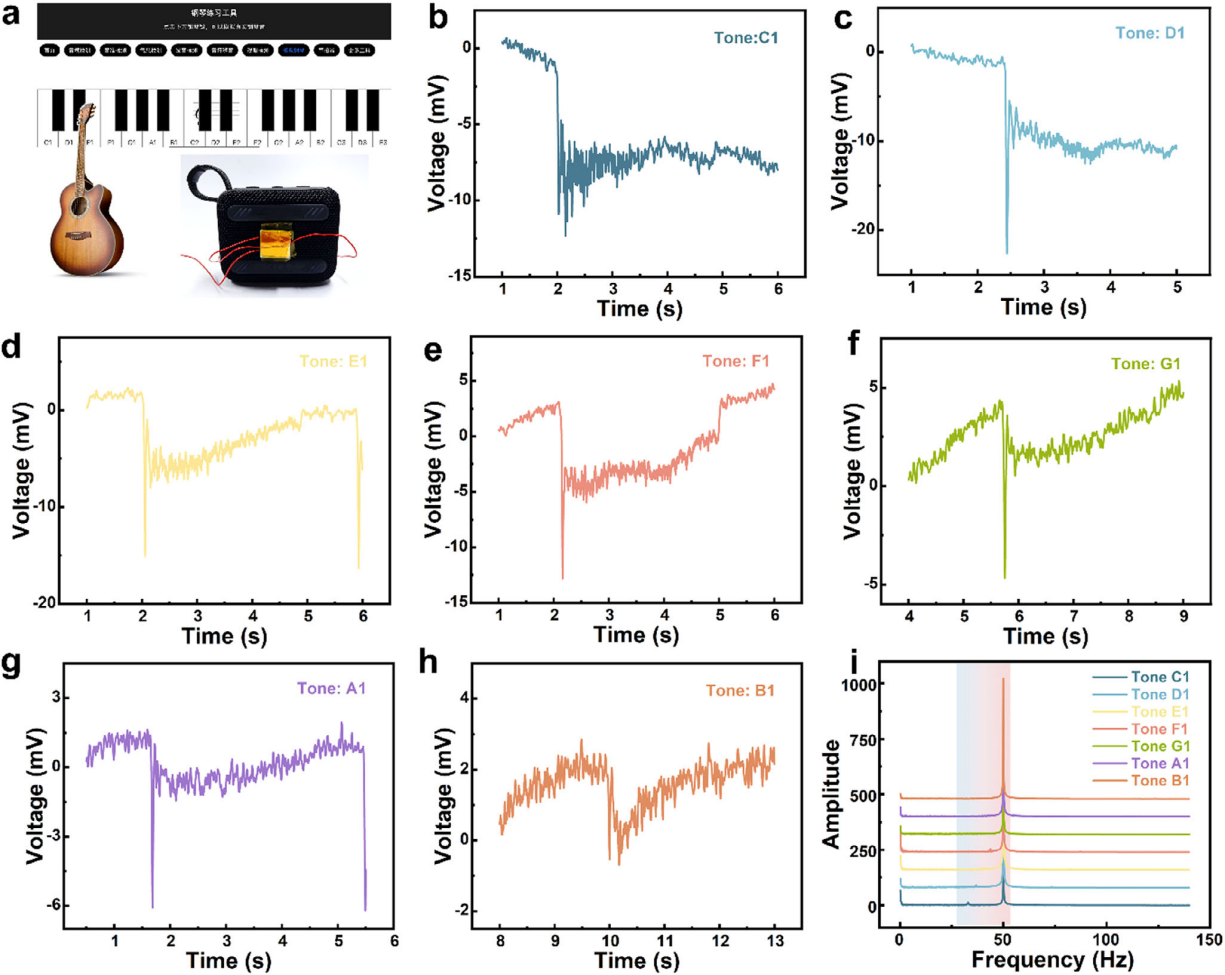
Tone recognition of musical instruments by the MXene/CNF aerogel@PDMS‐based dual‐modal pressure sensor. a) Interface and test equipment for simulating piano. Triboelectric voltage signals of electronic analog piano tones b) C1, c) D1, d) E1, e) F1, f) G1, g) A1, and h) B1. i) Triboelectric voltage output FFT signal of tones C1‐B1.

TPS has obvious advantages and potential in dynamic pressure stimuli monitoring, so we attach the TPS to the Bluetooth audio surface (**Figure** **6**a) for tone recognition. Connect the mobile phone with the Bluetooth speaker, emit different tones of C1‐B1 through the piano simulation software, and adjust the volume through the Bluetooth audio volume key. As shown in Figure 6b–h, different tones have different TPS voltage waveforms. In addition, by performing a simple Fourier transform on the voltage signal through the plotting software, it can be seen that the signals with different tones have different frequency peaks (Figure 6i). In addition, we assess the comprehensiveness of tone monitoring by testing the current output of the TPS at different tones. As shown in Figure S13a, Supporting Information, the sustained response times of the voltage and current waveforms of the TPS for the same tone C1 are consistent. We then monitored the current signal of C1–B1 tones, and the TPS current signal distinguished different tones very well (Figure S13b, Supporting Information). In addition, by adjusting the sound volume (Figure S13c, Supporting Information), the amplitude of the current signal also increased with increasing volume (Figure S13d, Supporting Information).

## 3 Conclusion

In summary, we designed and developed a dual‐modal pressure sensor by sharing the bottom electrode space and using MXene/CNF aerogel@PDMS simultaneously as a triboelectric‐piezoresistive sensing material. The excellent compressibility, recoverability, and fatigue resistance of the MXene/CNF aerogel@PDMS composite provide strong mechanical support for the dual‐modal pressure sensor. This enables the dual‐modal pressure sensor to have good sensing performance, high sensitivity (26.95 kPa^−1^ of TPS and 167.67 kPa^−1^ of PPS), rapid response and recovery times (0.3/0.4 ms of TPS and 4.71/2.99 ms of PPS), a maximum pressure detection of 183 kPa and an ultralow detection limit of 0.00346 kPa, and fatigue resistance over 3000 cycles. Most importantly, through the integration of dynamic triboelectric and static piezoresistive pressure sensing in longitudinal space with the same material, we finally achieved a variety of monitoring methods for complex signals, effective monitoring of the application and change of pressure stimuli, and good performance in human movement and physical signal monitoring.

## 4 Experimental Section

### Materials

Ti_3_AlC_2_ powder (99.8%, 200 mesh) was purchased from Jilin Eleven Technology Co., Ltd. (China). LiF (AR, 99.0%) was purchased from Shanghai Aladdin Biochemical Technology Co., Ltd. (China). Hydrochloric acid (AR) was purchased from Yonghua Chemical Co., Ltd. (China). The wood pulp paper used was bleached coniferous wood pulp paper purchased from Yangrun Trading (China). 2,2,6,6‐Tetramethylpiperidinooxy (TEMPO, AR, 97%) was purchased from Shao Yuan Technology (China). NaClO (CP, available chlorine 5.5–6.5%) was purchased from Shanghai Meryl Chemical (China). NaBr (99.99%) was purchased from Shanghai McLean Biochemical (China). NaOH (GR, 98%), polyvinyl alcohol (PVA, Type 1799, AR), and n‐hexane (AR, 97.0%) were purchased from Shanghai Titan Technology (China). PTFE granules (5 µm, specific surface area > 7.5m^2^ g^−1^) were purchased from West Asia Chemical Co., Ltd. (China). Polydimethylsiloxane (PDMS) was purchased from Dow Chemical Company (USA). Silicone rubber (Ecoflex 00–50) was purchased from Smooth‐On (USA).

### Preparation of Cellulose Nanofiber (CNF)

Cellulose nanofibers were fabricated via TEMPO chemical oxidation and physical ultrasonic crushing methods.^[^ ^35^ ^]^ First, 1 g of wood pulp paper was dispersed in 400 mL of water. After the suspension was stirred thoroughly, 0.033 g of TEMPO oxidizer, 0.33 g of NaBr, and 20 mL of NaClO were added. HCl and NaOH solutions (0.5 m) were used to ensure that the pH of the wood pulp suspension fully reacted for 10 h in the range of 10–10.5. After the excess impurities in the suspension were washed with water, the fiber suspension was broken by ultrasonication. Finally, the clarified cellulose nanofiber suspension was obtained and configured into a 10 mg mL^−1^ suspension.

### Synthesis of MXene Sheets

MXene nanosheets were prepared by etching Ti_3_AlC_2_ powder with LiF/HCl. The etching solution was obtained by dissolving LiF (1.6 g) in 12 m HCl (20 mL) and slowly adding 1 g of Ti_3_AlC_2_ powder. The MXene suspension was washed after 24 h of reaction in a 45 °C water bath. First, the supernatant of the MXene suspension was removed by centrifugation at 3500 rpm for 1 min. Then, the excess LiF in the MXene solution was washed with 2 m HCl. Finally, the MXene suspension was washed with water until its pH exceeded 5. After 1 h of ultrasonic treatment with nitrogen, the MXene suspension was centrifuged at 3500 rpm for 20 min to obtain a uniform supernatant containing MXene sheets.

### Preparation and Regulation of the MXene/CNF Aerogel

First, 5 mL of a 10 mg mL^−1^ CNF suspension was supplemented with a 1:1 crosslinker (polyvinyl alcohol (PVA) solution). After the CNF/PVA solution was heated and stirred at 60 °C for 2 h, 5 mL of the MXene suspension (wt.%, 100 mg of MXene) was added, and the mixture was stirred at room temperature to ensure that the CNFs and MXene were evenly dispersed. The homogeneous mixture was placed into a silicone mold and frozen for setting. After the samples were completely frozen, the ice crystals were removed by sublimation after vacuum freezing for >60 h at −80 °C. The freeze‐dried aerogel was unmolded and immersed in a PDMS solution (10 wt.%) diluted with hexane for 10 s and then dried in an oven at 60 °C for 10 h. The treated aerogel was named aerogel@PDMS. By changing the content of MXene and CNF, mixed solution concentrations of 10, 15, and 20 mg mL^−1^ aerogel were obtained. For convenience, the samples were designated aerogel‐10, aerogel‐15, and aerogel‐20. The MXene aerogel was prepared in the same way as the MXene/CNF aerogel as the reference sample.

### Characterization of the MXene/CNF Aerogel@PDMS

The MXene sheets were characterized via transmission electron microscopy (Thermo Fisher Scientific, FEI Talos F200X). The aerogel microstructure was characterized via field emission scanning electron microscopy (Hitachi, SU8230). X‐ray diffraction (XRD) patterns were obtained via a PANalytical X‐ray powder diffractometer. A universal material testing machine (34TM‐50, 5 mm min^−1^) was used to test the mechanical properties of the samples.

### Fabrication of the Dual‐Modal Pressure Sensor

First, the MXene/CNF aerogel@PDMS worked as the tribo‐layer of TPS and the force‐sensitive layer of PPS. The contact separation mode was used for the TPS, and the other tribolayer used PTFE particles (5 µm) mixed with silicone rubber (00‐50). Copper was selected as the electrode, PMMA was used as the base, and a polycarbonate film was used as the outer packaging material. The PPS had two electrodes on the same side, sharing the bottom space of the aerogel with one of the triboelectric electrodes.

### Performance Characterization of the Dual‐Modal Pressure Sensor

Using a linear motor (E1200) to test triboelectric output performance, software was used to control the contact‐separation frequency. The pressure applied to the sensor was controlled by a manometer (DFS‐II, Chatillon). High‐frequency pressure signals are provided with a vibration exciter. All triboelectric voltage signals are measured by a programmable electrometer (Keithley 6514), and real‐time data acquisition and analysis are realized via the LabVIEW software platform. With a Keysight B2901A source meter, the voltammetry characteristics of the aerogel were measured in the voltage range of −1 to 1 V. The current signal of the piezoresistive pressure sensor was measured with a Keithley 2400 source meter (1 V external voltage), and the resistance signal was measured with a programmable electrometer (Keithley 6514).

## Conflict of Interest

The authors declare no conflict of interest.

## Supporting information



Supporting Information

## Data Availability

The data that support the findings of this study are available from the corresponding author upon reasonable request.
