# Supplementary material for: Superelastic and Ultra‐Soft MXene/CNF Aerogel@PDMS‐Based Dual‐Modal Pressure Sensor for Complex Stimuli Monitoring
Source: Adv Sci (Weinh). 2025 Apr 7;12(26):2502797. doi: 10.1002/advs.202502797 (PMC12244993; doi:10.1002/advs.202502797)
Supplement: Supplementary file 1 — Supporting Information [file ADVS-12-2502797-s001.docx]

Supporting Information

Superelastic and Ultra-Soft MXene/CNF Aerogel@PDMS-based Dual-Modal Pressure Sensor for Complex Stimuli Monitoring

Ao Wang, Zhenqiu Gao, Shaokuan Wu, Yihan Wei, Bohan Lu, Jia Shi, Lanyue Shen, Yina Liu, Xuhui Sun and Zhen Wen*

**Supplementary Note S1**

**Calculation method for the compression hysteresis energy of aerogels**

Compression rebound test of aerogels was performed in compression mode with a universal material testing machine. According to the obtained loading and unloading curves, the area formed by the two curves is integrated by Origin software to obtain the compressive hysteresis energy. The specific formula is as follows:

$$H=\int_{0}^{x} \left( y_{loading}-y_{unloading} \right)dx$$

Where *H* is the hysteresis energy, *x* is the corresponding maximum compressive strain degree, and *y*_loading_ and *y*_unloading_ are the loading and unloading curves respectively.


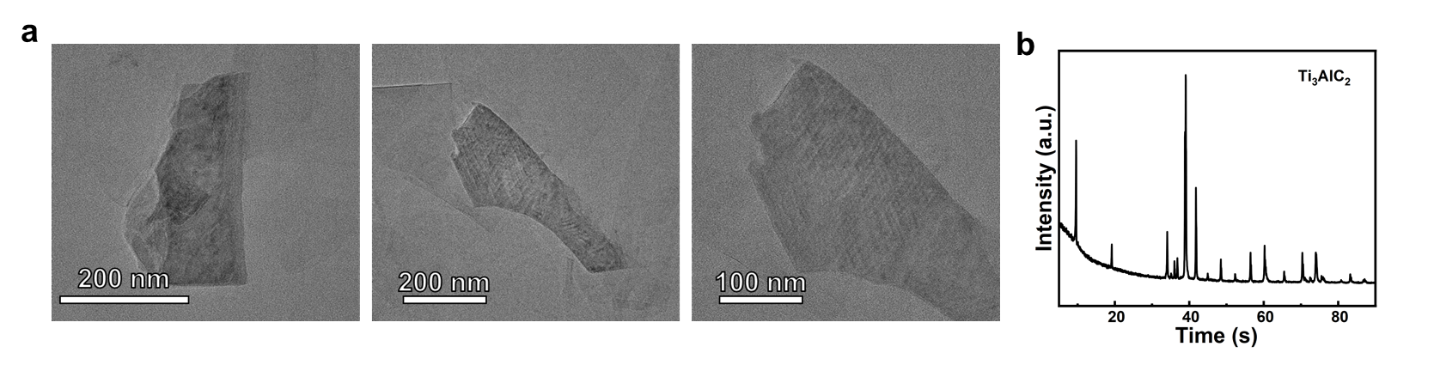


**Figure S1**. **Characterization of MXene.** (a) TEM image of MXene nanosheets. (b) XRD spectra of Ti_3_AlC_2_.


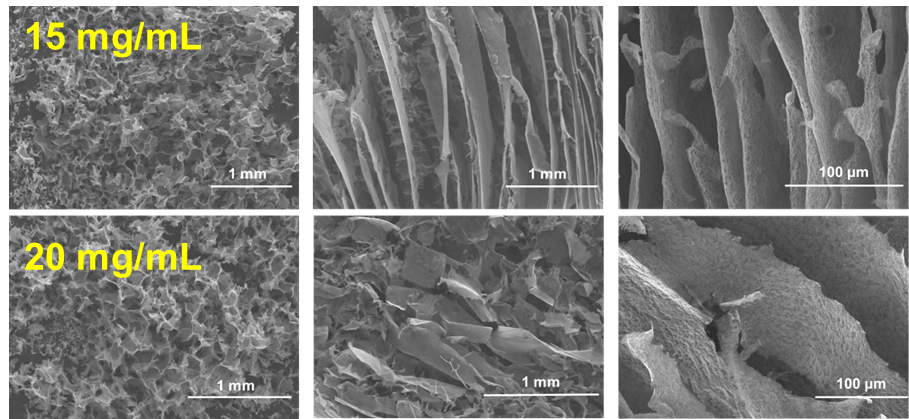


**Figure S2.** **SEM of aerogel-15 and aerogel-20.**


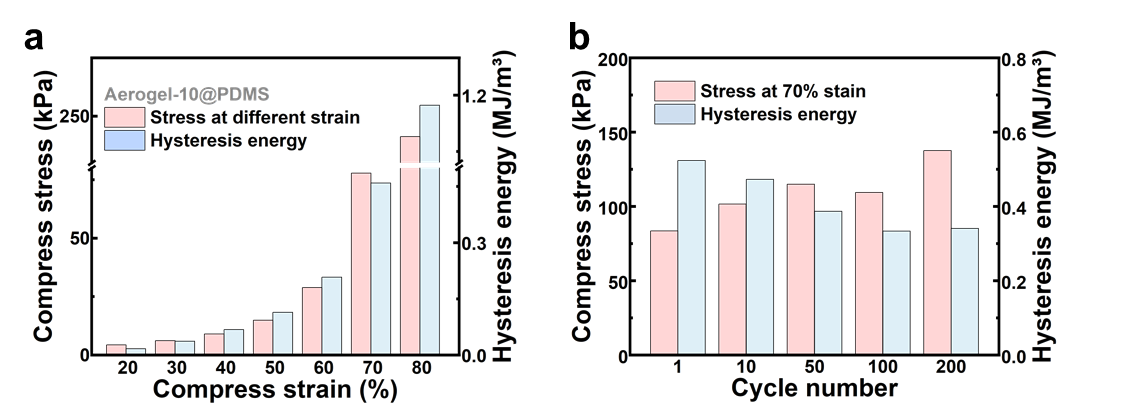


**Figure S3.** **Hysteresis energy and stress analysis of aerogel compression.** (a) Compressive stress and hysteresis energy of aerogel-10@PDMS at 20–80% strain. (b) Compressive stress and hysteresis energy of aerogel-10@PDMS at 70% strain during the 200th cycle.

**
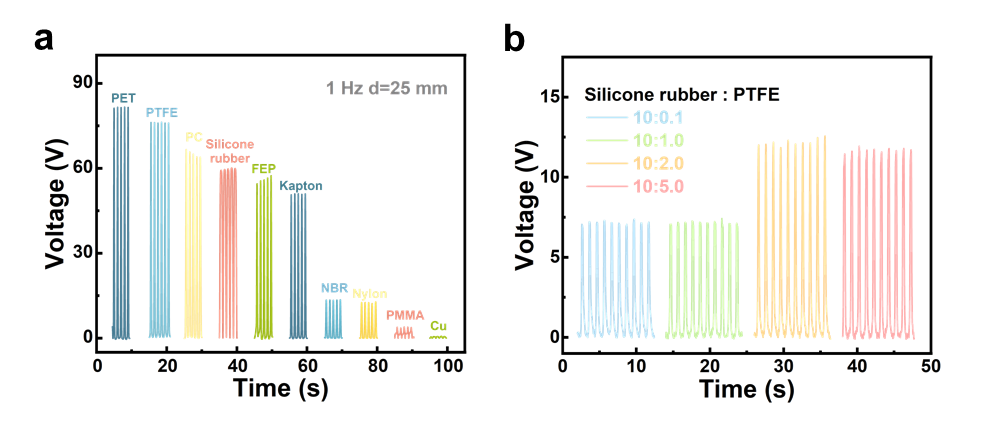
**

**Figure S4. Tribolayer material selection of TPS.** (a) Voltage output of aerogel-10@PDMS tribo with different materials. (b) Voltage output of aerogel-10@PDMS tribo with various ratios of silicone rubber@PTFE.


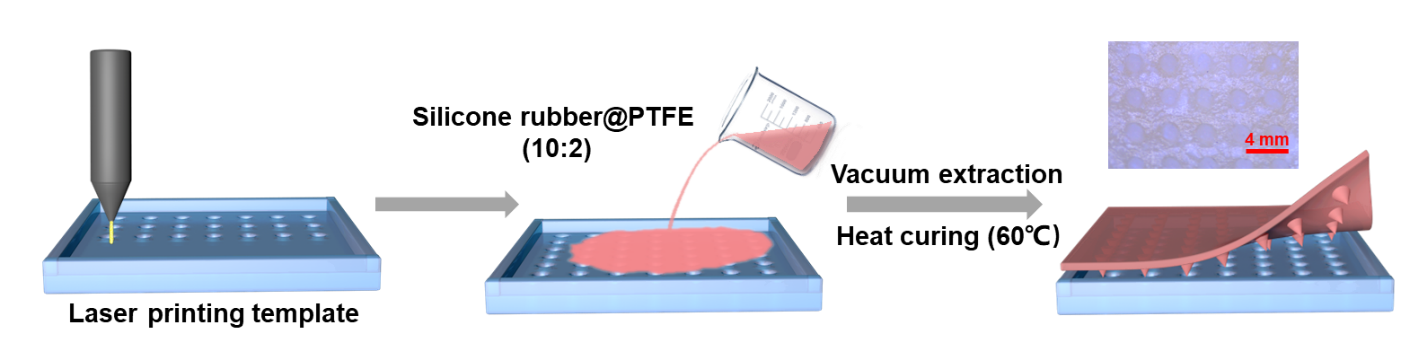


**Figure S5.** **Preparation and optical photograph of silicone rubber@PTFE.**


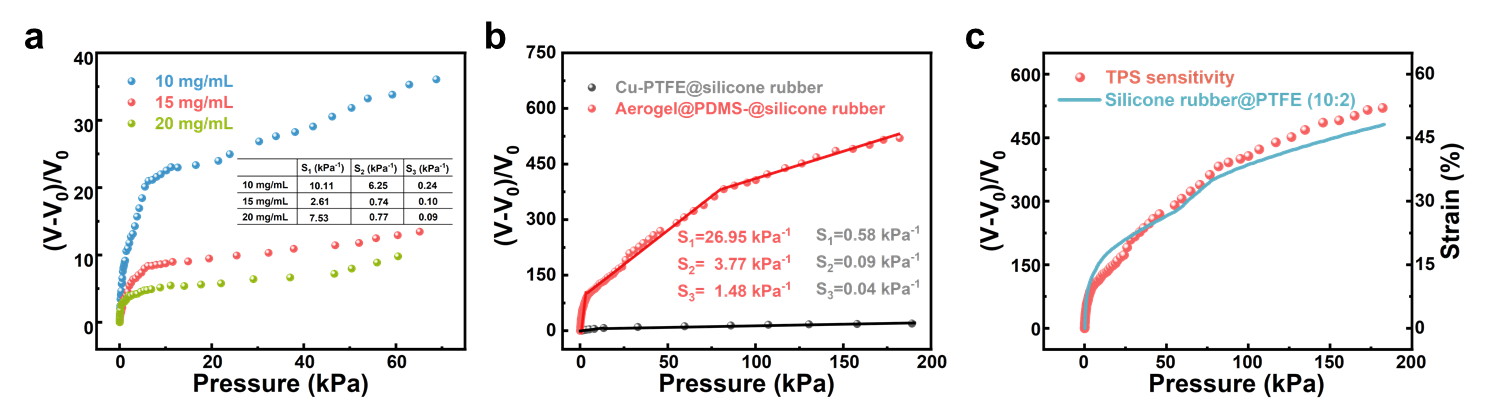


**Figure S6.** **Sensitivity of TPS for different tribolayers.** (a) Triboelectric pressure sensing sensitivity of various concentrations of aerogel. (b) Triboelectric pressure sensing sensitivity of aerogels or Cu tribolayers. (c) Relationship between the triboelectric sensitivity and mechanical properties of silicone rubber@PTFE.


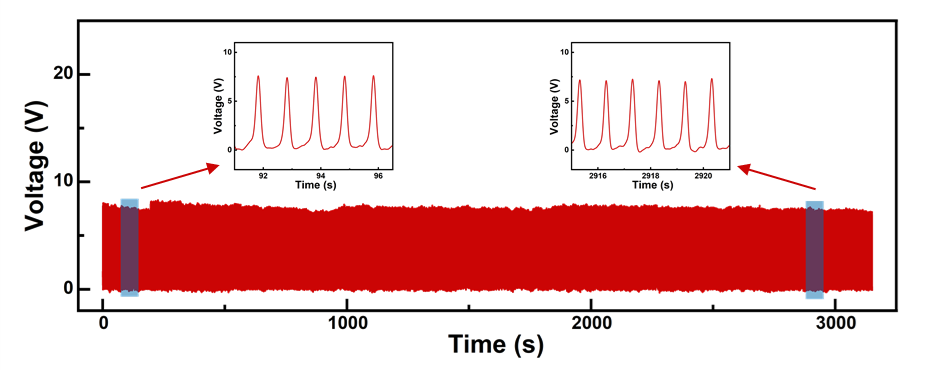


**Figure S7.** **Triboelectric property stability after aerogel placed in air for 20 days.**


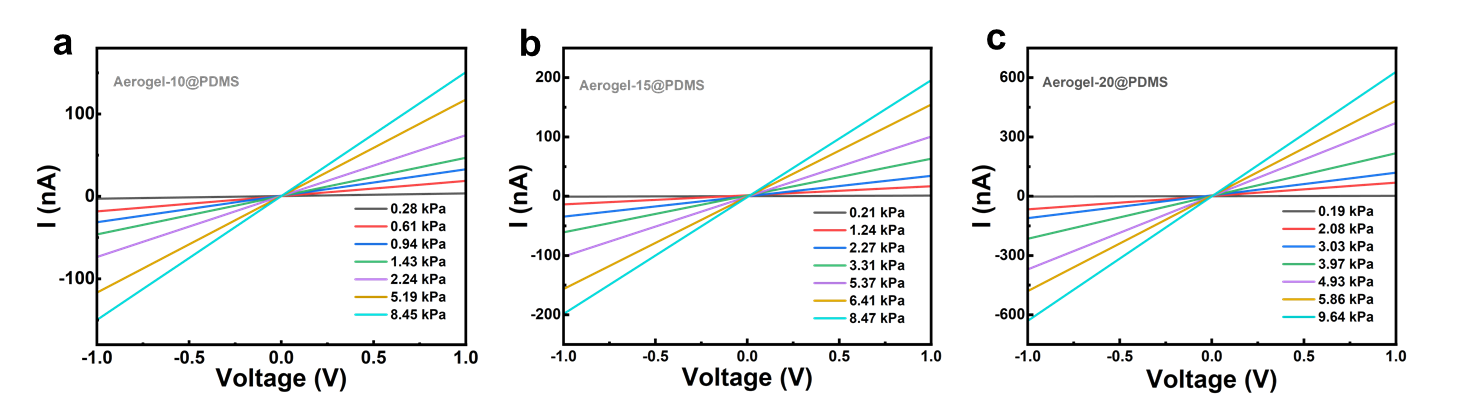


**Figure S8.** **Volt‒ampere characteristic curve.** (a) Aerogel-10@PDMS. (b) Aerogel-15@PDMS. (c) Aerogel-20@PDMS.


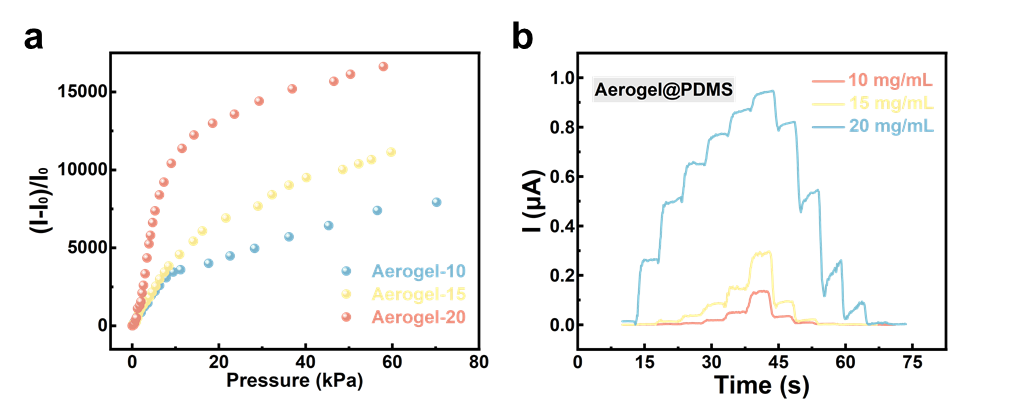


**Figure S9. Piezoresistive properties of different aerogels.** (a) Piezoresistive sensitivity of various concentrations of aerogel. (b) Piezoresistive static force tests of various concentrations of aerogel.


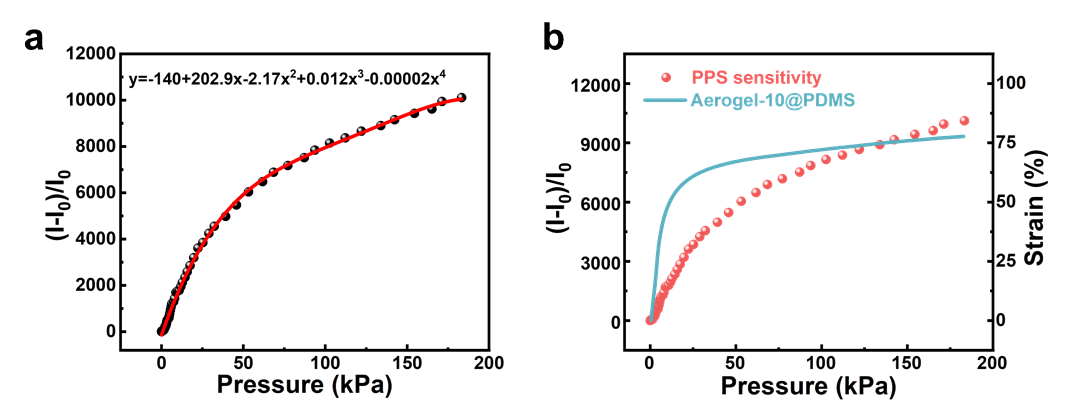


**Figure S10.** **Piezoresistive sensitivity curve analysis of aerogel-10@PDMS.** (a) Piezoresistive sensitivity polynomial fitting curve. (b) Relationship between piezoresistive sensitivity and mechanical properties of aerogel.


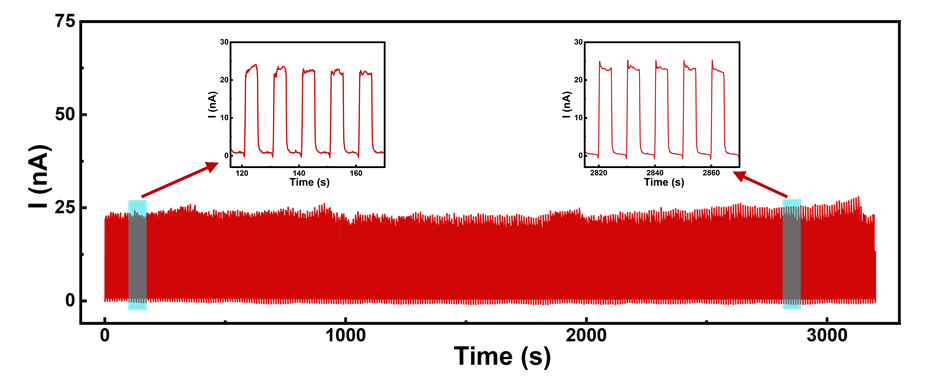


**Figure S11.** **Piezoresistive property stability after aerogel placed in air for 20 days.**


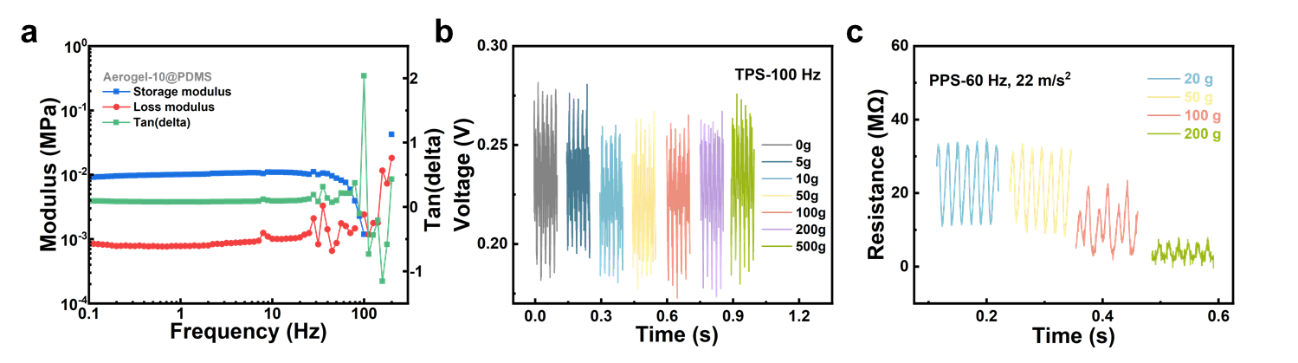


**Figure S12. High-frequency signal of the dual-modal pressure sensor.** (a) Dynamic mechanical analysis of MXene/CNF aerogel-10@PDMS. (b) 100 Hz signal of the triboelectric pressure sensor under different prestresses. (c) 60 Hz signal of the piezoresistive pressure sensor under different prestresses.


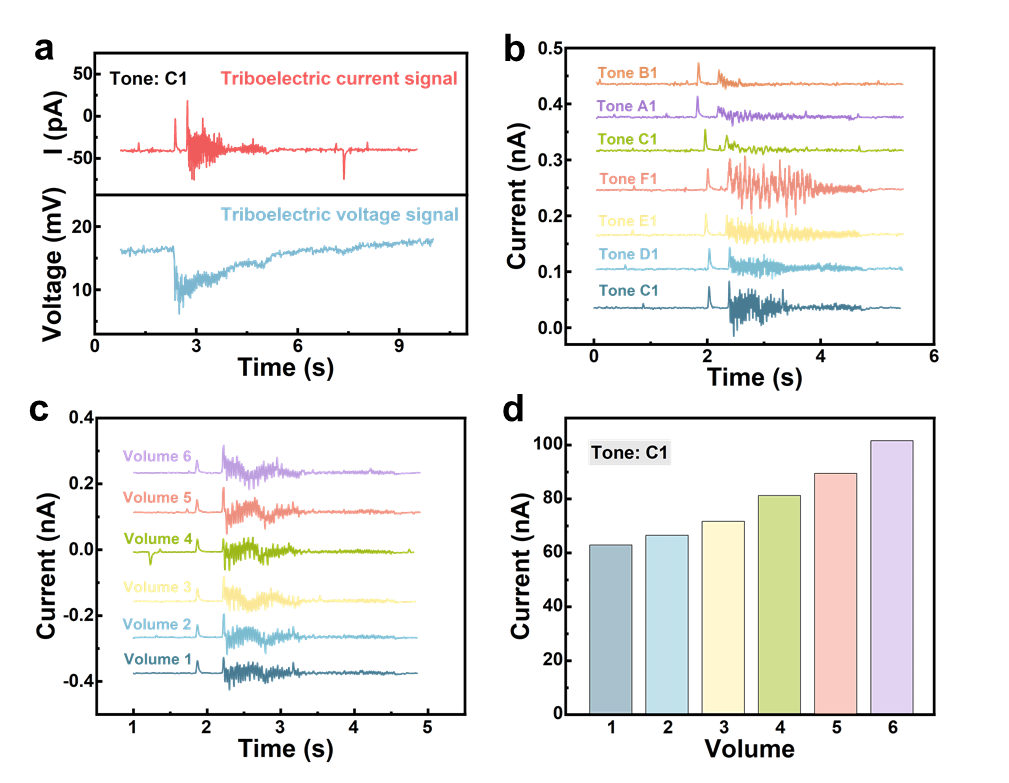


**Figure S13. TPS current signal of the dual-modal pressure sensor for tone recognition.** (a) Voltage and current output contrast of tone identification. (b) Current output of identification of tone C1--B1. (c-d) Current output of identification of tone C1 at different volumes.
